# Self-Assembled Modified Soy Protein/Dextran Nanogel Induced by Ultrasonication as a Delivery Vehicle for Riboflavin

**DOI:** 10.3390/molecules21030282

**Published:** 2016-03-15

**Authors:** Bei Jin, Xiaosong Zhou, Xiangzhong Li, Weiqin Lin, Guangbin Chen, Riji Qiu

**Affiliations:** 1 School of Chemistry and Chemical Engineering, Lingnan Normal University, Zhanjiang 524048, China; zxs801213@163.com (X.Z.); lixiangzhong12@163.com (X.L.); linweiqin12@sina.com (W.L.); byrlxz@163.com (G.C.); qiuriji12@126.com (R.Q.); 2 Engineering Research Center of Starch and Vegetable Protein Processing, Ministry of Education, South China University of Technology, Guangzhou 510640, China

**Keywords:** soy protein, heat denaturation, alcalase hydrolysis, dextran, nanogels, ultrasonication, self-assembly

## Abstract

A simple and green approach was developed to produce a novel nanogel via self-assembly of modified soy protein and dextran, to efficiently deliver riboflavin. First, modified soy protein was prepared by heating denaturation at 60 °C for 30 min or Alcalase hydrolysis for 40 min. Second, modified soy protein was mixed with dextran and ultrasonicated for 70 min so as to assemble nanogels. The modified soy protein-dextran nanogels were characterized by Fourier-transform infrared spectroscopy (FTIR) and X-ray photoelectron spectroscopy (XPS) and ζ-potential studies to confirm the formation of NGs. Transmission electron microscopy (TEM) revealed the NGs to be spherical with core-shell structures, in the range of 32–40 nm size. The nanogels were stable against various environmental conditions. Furthermore, the particle size of the nanogels hardly changed with the incorporation of riboflavin. The encapsulation efficiency of nanogels was found to be up to 65.9% at a riboflavin concentration of 250 μg/mL. The nanogels exhibited a faster release in simulated intestine fluid (SIF) compared with simulated gastric fluid (SGF). From the results obtained it can be concluded that modified soy protein-dextran nanogels can be considered a promising carrier for drugs and other bioactive molecule delivery purposes.

## 1. Introduction

Nanogels are three-dimensional, nanosized hydrogel particles with many attractive properties like small particle sizes, high water uptake, high mechanical stability, excellent loading capacity for bioactive compounds, controlled release, smart responses to environmental stimuli. This means they have the potential to become an ideal bioactive compound delivery system [1,2]. Natural biopolymer-based nanogels have gained more and more attention as delivery systems due to their biodegradability, biocompatibility, and similarity to the macromolecular components of the extracellular matrix [3]. In addition, many biopolymers such as polysaccharides and proteins possess a large amount of functional groups which can be utilized for further bio-conjugation, allowing hydrogel performance to be adapted to a specific application. Various polysaccharides and proteins have been used for fabricating stable nanogels [4,5,6]. These studies showed that the size, charge, and stability of the biopolymer particles formed depend on the pH, ionic strength, ratio of protein to polysaccharide, biopolymer type, heating time and temperature [7,8,9].

Soy protein is a natural polymer widely tested as a starting material for the synthesis of polymeric networks to be used in bioactive compounds delivery due to its natural abundance, desirable water solubility, good biocompatible and biodegradable, and non-immunogenic and anti-carcinogenic properties [10]. Dextran is well known for its degradability by dextranase, biocompatibility and non-toxicity, and as a result has been used over the last two decades for the encapsulation of macromolecules [11]. Therefore, it seems that soy protein and dextran are ideal candidates for preparing bioactive compound nanocarriers. We have previously developed a simple and green method to fabricate nanoparticles with dextran-conjugated soy protein by TiO_2_ photocatalysis [12]. However the particle size of the nanoparticles was too large to deliver functional materials with longer circulation time *in vivo*. Feng and co-workers prepared stable nanogel by simply heating soy β-conglycinin-dextran conjugates [13], but little research on loading and unloading bioactive substance in soy β-conglycinin-dextran nanogels was conducted, which may limit these nanogels in terms of use in bioactive substance delivery system. 

Herein, a simple and effective strategy for the fabrication of biodegradable nanogels with smaller particle sizes due to the use of ultrasonication as bioactive compound nanocarriers is presented. The structure and stability of nanogels were characterized by various physicochemical techniques. Riboflavin was used as the model drug to evaluate the loading, physicochemical properties and release profile of soy protein/dextran complex nanogels due to low molecular mass, partially water solubility, easy diffusion and quantitative analysis [14]. This study would be helpful to the future design of more effective and safer bioactive substance delivery-carrier systems.

## 2. Results and Discussion

### 2.1. Optimization of the Fabrication of Modified Soy Protein/Dextran Nanogels

**Figure 1:**
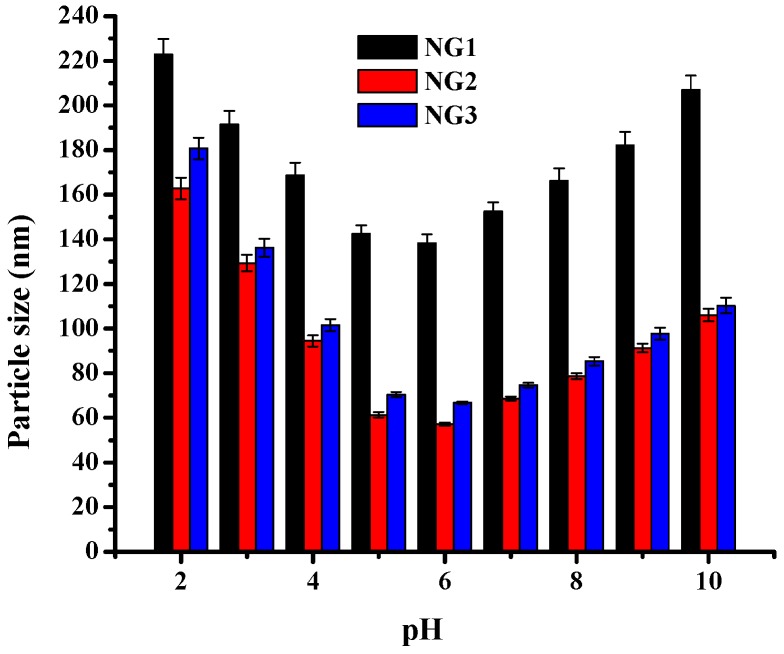
The particle sizes of NG1, NG2 and NG3. The NGs were prepared by ultrasonicating soy protein-dextran mixtures at different pH values for 70 min. Data with different lowercase letters in the same test are significantly (*p* < 0.05) different.

**Figure 2:**
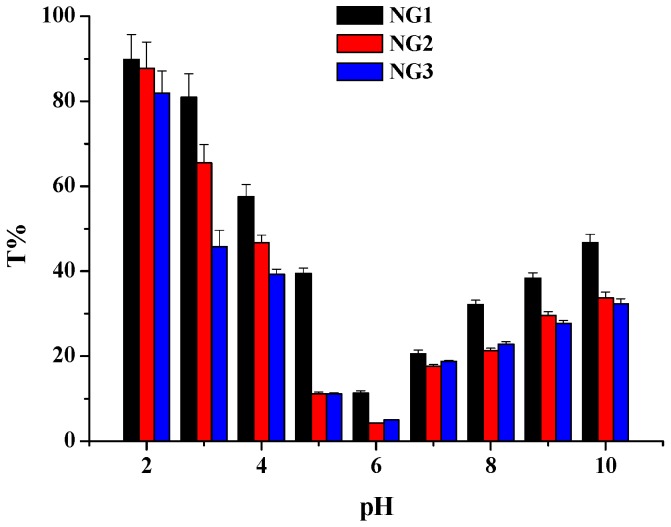
Presentation of the turbidity of NG1, NG2 and NG3. The NGs were prepared by ultrasonicating soy protein-dextran mixtures at different pH values for 70 min. Data with different lowercase letters in the same test are significantly (*p* < 0.05) different.

The sizes of different blank nanogels were characterized by DLS. Figure 1 shows the influence of pH on the particle size of the nanogels, which were formed by ultrasonicating modified soy protein and dextran mixed solutions (weight ratio of 1:1, ultrasonicated for 75 min) at different pH. When the pH dropped from 10 to 6, the average size of all formulations nanogels obviously decreased. In the pH range of 6–5, the mean particle size of the NGs in all formulations changed slightly. At pH∼pI, the modified soy protein tended to remain associated with dextran during the ultrasonication process, which inhibited the soy protein molecules from forming large aggregates. As the pH was decreased further, the average size and PDI of the NGs increased sharply. This is because physical interactions such as electrostatic attraction between modified soy protein and pectin were relatively strong, and large aggregates either remain or were formed after ultrasonication. The turbidity of these nanogels as a function of pH was also investigated. As shown in Figure 2, the turbidity observed almost a similar trend. Correspondingly, turbidity decreased remarkably from pH 2 to 5, but mildly decreased from pH 5 to 6, and then increased obviously as pH increased in all the samples. This resulted in coalescence of the NGs and led to the formation of larger sizes. All formulations of the NGs exhibited a unimodel size distribution near pH 6, suggesting that the polymerization of these NGs induced by ultrasound could offer good control over the size. Therefore, pH 6 was chosen as the optimal pH value for nanogel formation in the following study.

### 2.2. Characterization of Modified Soy Protein/Dextran Nanogels

**Figure 3:**
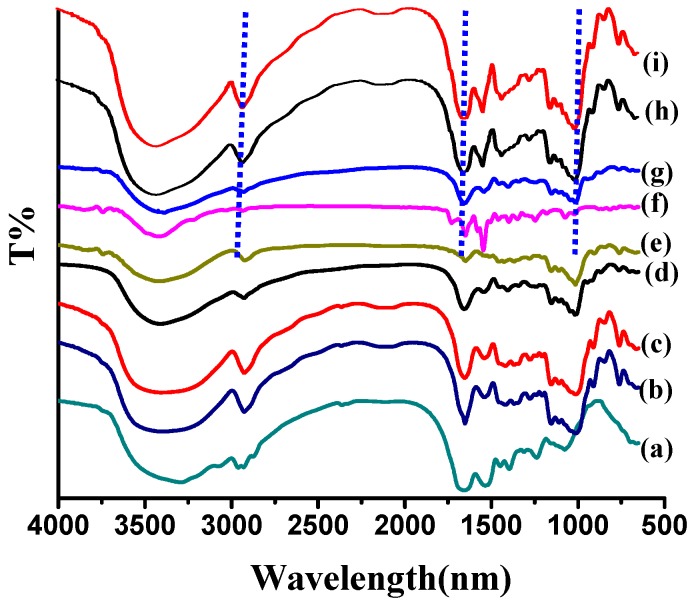
FTIR spectra of soy protein (**a**); NG1 (**b**); NG2 (**c**); NG3 (**d**); dextran (**e**); riboflavin (**f**); riboflavin-loaded NG3 (**g**); riboflavin-loaded NG2 (**h**) and riboflavin-loaded NG1 (**i**).

FTIR spectroscopy was used to confirm formation of nanogels. The FTIR spectra of soy protein, dextran and blank nanogels are shown in Figure 3.The IR spectrum peaks of pure soy protein at 3294, 1655 and 1535 cm^−1^ were assigned to the characteristic vibrations of OH, Amide I (C=O stretching) and Amide II (N–H bending and C–N stretching) bands, respectively [15]. As for dextran, the peaks obtained at 3418 cm^−1^, 2924 cm^−1^, 1180–953 cm^−1^ were those of OH stretching, C–H bending, C–O stretching and OH bending, respectively [16]. The blank nanogels (NG1, NG2 and NG3) showed the coincident characteristic absorption bands of soy protein and dextran with spectra of varying intensities, but these peaks that belong to double bonds completely disappeared, confirming the formation of co-polymeric NGs due to some physical interactions between them.

**Figure 4:**
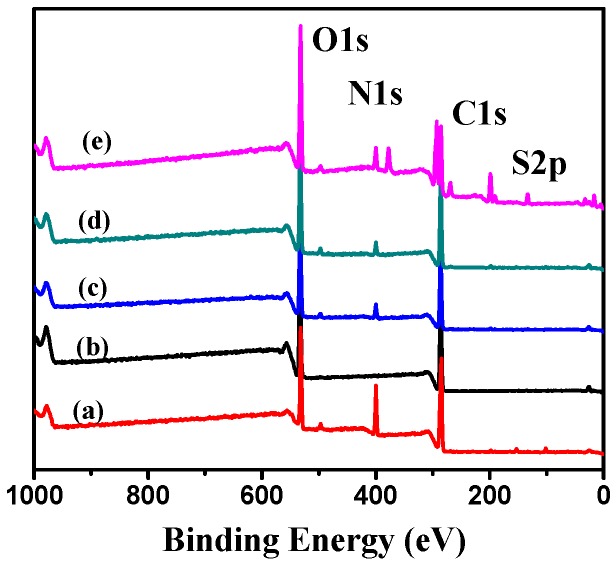
XPS peak fitting curves of (**a**) soy protein; (**b**) dextran; (**c**) NG1; (**d**) NG2 and (**e**) NG3.

Figure 4 shows the XPS wide-scan spectra of (a) soy protein; (b) NG1; (c) NG2; (d) NG3; and (e) dextran. A wide survey XPS spectrum of the blank nanogels mainly contained C 1s, O 1s and N 1s. The high energy resolution spectra of nanogels showed a peak at binding energy near 163 eV for S 2p. The C 1s, O 1s, N 1s and S 2p of NG1 contained 57.88%, 34.15%, 6.02% and 0.19%. The C 1s, O 1s, N 1s and S 2p of NG2 contained 57.99%, 35.67%, 4.17% and 0.22%. The C 1s, O 1s, N 1s and S 2p of NG3 contained 41.77%, 32.02%, 6.38% and 0.23% and 61.32%, 20.84%, 14.49% and 0.38% for soy protein. Obviously, the peaks of N 1s and S 2p, which only exist in soy protein, appeared in the spectrum of all nanogels with different proportion. Besides, N could be used as characteristic element of protein. The proportions of N 1s peak in all nanogels spectrum were markedly below the proportion of soy protein. Therefore, taking the zeta potential result into account, we can speculate that part of soy protein are supposed to be trapped in the center of nanogels after ultrasonicating through the physical interactions between soy protein and dextran, while dextran can mostly be distributed on the surface of the nanogels.

### 2.3. Morphology Characterization

**Figure 5:**
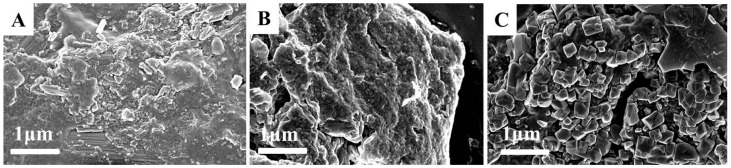
SEM images of NG1 (**A**); NG2 (**B**) and NG3 (**C**).

**Figure 6:**
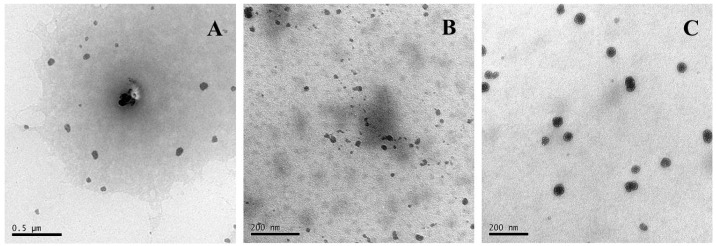
TEM images of NG1 (**A**); NG2 (**B**) and NG3 (**C**).

To shed more light on the modified soy protein/dextran nanogels, the morphology of the soy protein particles was observed by TEM and SEM performed on the resulting samples (Figure 5 and Figure 6). The SEM images of all formulations NG particles were recorded and showed the presence of a rough surface and irregularly shaped and discrete particles which may be formed during the drying process. This is because when the water molecules on the conductive adhesive are evaporated, the modified soy protein/dextran nanogels will congregate together. The image also shows that the size of all formulation nanogels were greater than 200 nm.

To further investigate the morphology in the nanoscale distribution, the TEM images of the NG1 particles were recorded and the images showed the presence of near spherical shapes, and some particle aggregates exist. The average size of NG1 was 89 ± 5 nm. However, the blank nanogels (NG2 and NG3) particles were well dispersed with a regular spherical shape having core-shell like structure. Thermal denaturation and enzymatic hydrolysis resulted in the exposure of some active groups in soy protein and promoting the interaction between soy protein and dextran, which was in favorable of the formation of core-shell like structure. This result was in accordance with Feng *et al.* [13] who reported the dextran attached to the β-conglycinin forming and hydrophilic dextran chains constituted the dark shell region whereas the hydrophobic cross-linked β-conglycinins constituted the light core region. From the TEM image, the average diameter of NG2 and NG3 particles were about 32 ± 10 nm and 40 ± 10 nm, respectively, which was smaller than that obtained from DLS analysis. The reason for this is that the nanogels measured by DLS were hydrated whereas those measured by TEM were dehydrated [17]. These results were also in accordance with the above XPS analysis. In our study, the modified soy protein/dextran nanogels had a similar average mean diameter of approximately 32–40 nm. Therefore, we found that ultrasonication is appropriate in preparing soy protein/dextran nanoparticles with a rather uniform size distribution which could cause soy protein to further unfold and interact with dextran as well as promote aggregates and form nanogels.

### 2.4. Physical Stability of Nanogels

**Figure 7:**
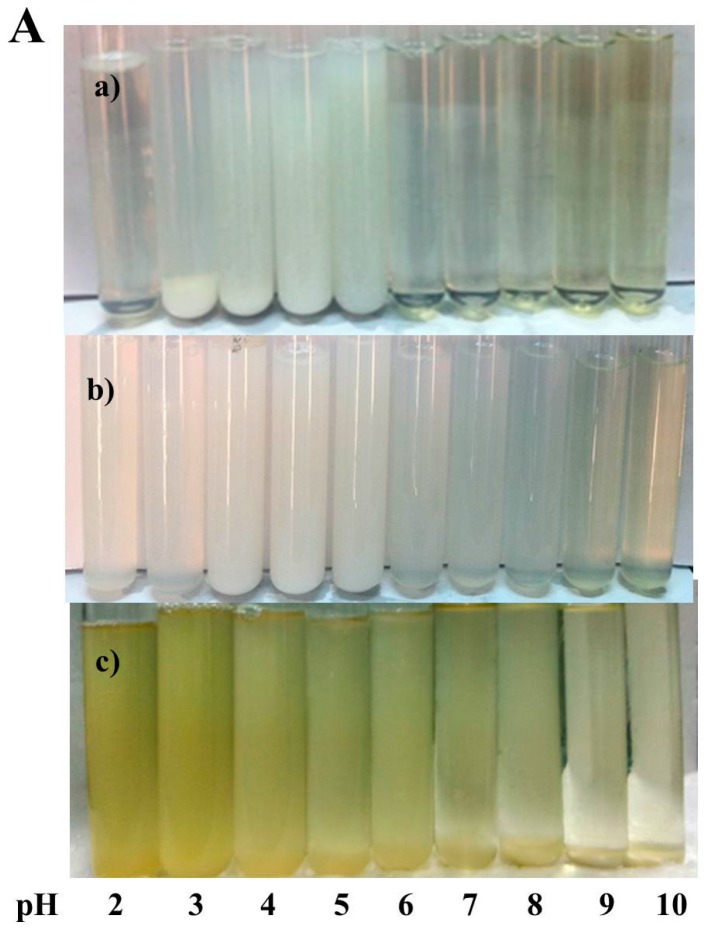

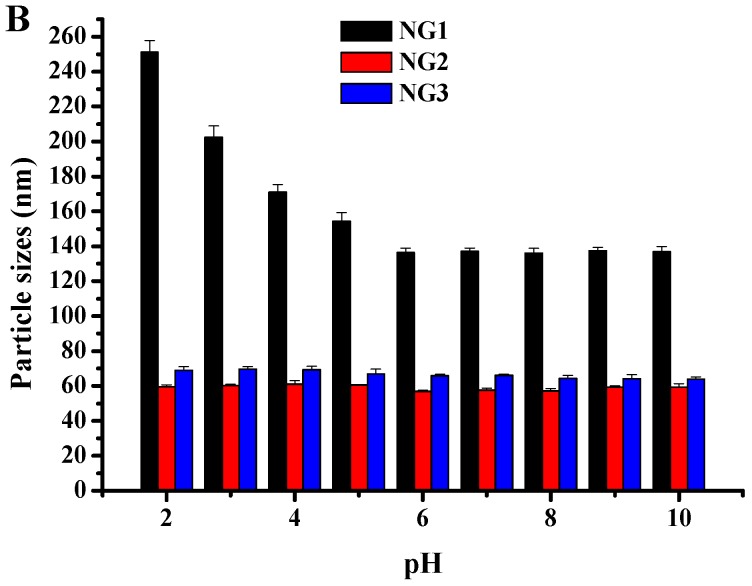
Effects of pH on visual appearance (**A**) and the particles sizes (**B**) of NG1 (**a**); NG2 (**b**) and NG3 (**c**). The data with different lowercase letters in the same test are significantly (*p* < 0.05) different.

The nanogels were incubated at various pH values to investigate the stability. The optical photograph of the nanogels (NG1, NG2 and NG3) as a function of pH is showed in Figure 7A. NG1 and NG 2 presented transparent and colorless, but NG3 showed light yellow and transparent. NG1 was unstable in the pH range of 2–6 as secondary aggregation of protein particles happened and the particles precipitated. As such, there are no obvious changes in particle sizes of NG1 when the pH value is above 6.0 as shown in Figure 7B. A substantial increase in particle size was first observed around pH 5.0, and a further increase of diameters caused extensive particle sedimentation as pH dropped. This phenomenon caused by weakening of electrostatic interaction among nanogels as pH decreased. However, the nanogel (NG2 and NG3) solutions were homogeneous in appearance (Figure 7A), and particle sizes did not change significantly after incubation in media of pH 2.0−10.0 (Figure 7B). This could be mainly due to the core-shell like structure of nanogels, where hydrophilic dextran shell provided hydrophilicity and steric hindrance in aqueous media that can protect the soy protein from precipitation. The results were in accordance with those of Ding *et al.,* who fabricated soy protein/soy polysaccharide complex nanogels via self-assembly [6].

**Figure 8:**
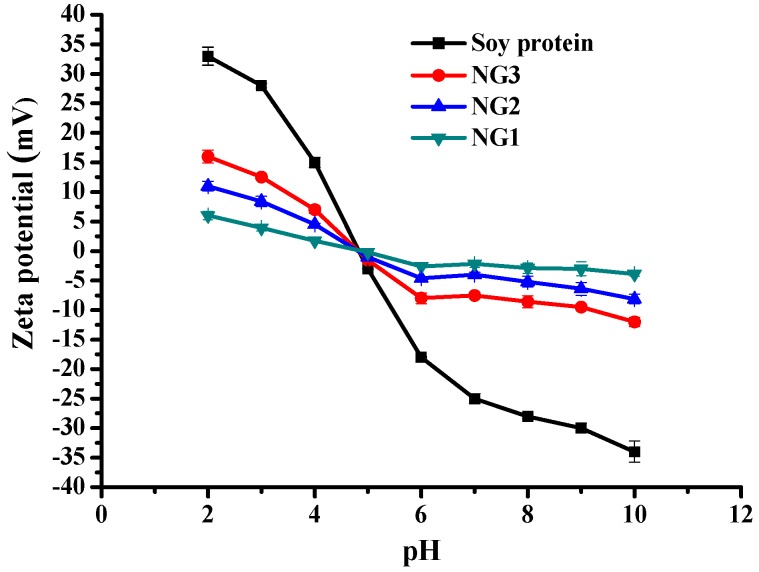
Effects of pH on ζ-potential of soy protein, NG1, NG2 and NG3. The data with different lowercase letters in the same test are significantly (*p* < 0.05) different.

The ζ-potential of soy protein and all nanogels at different pH values was also monitored at different pH values (Figure 8). The ζ-potential of nanogels was pronouncedly lower in magnitude compared with native soy protein and that the downward trends were different from those of soy protein, which were in accordance with previous report of soy protein/soy polysaccharide, soy β-conglycinin/dextran and β-lactoglobulin/pectin nanogels with core-shell like structure [6,13,18]. Changing the medium pH from 6 to 10 caused a strong electrostatic interaction to occur in modified soy protein and dextran, which was improved nanogel stability. These results further confirmed the above FT-IR and XPS analysis. Moreover, NG3 carried more charges which indicated that more polysaccharide chains on the NG3 surface were extended, resulting in larger particle sizes than those of NG2.

**Figure 9:**
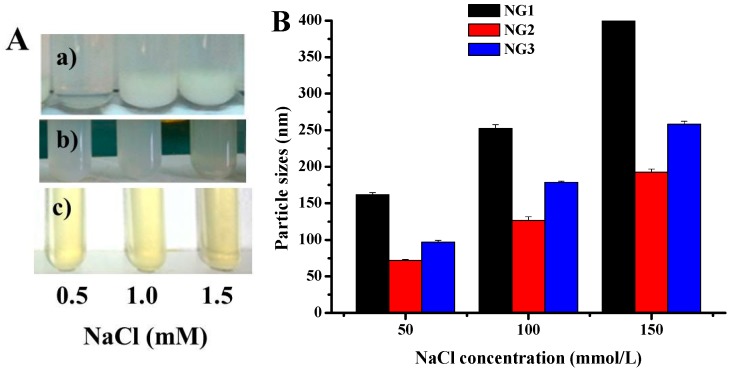
Influences of NaCl concentration on visual appearance (**A**) and the particles sizes (**B**) of NG1 (**a**); NG2 (**b**) and NG3 (**c**). The data with different lowercase letters in the same test are significantly (*p* < 0.05) different.

The effect of NaCl concentration on the stability of nanogels was also investigated (Figure 9). When the NaCl concentration reached to 150 mM, the particle sizes of NG1 increased to above 0.5 μm with a little precipitation, but no precipitation occurred in modified soy protein/dextran nanogels (Figure 9A). As such, the particle sizes of all formulation nanogels had a dramatic increase when the NaCl concentration increased to 150 mM (Figure 9B). It is possible that, in the presence of NaCl, intermolecular electrostatic forces were modified. Moreover, this electrostatic charge screening effect of NaCl might facilitate the molecular association between soy protein and dextran. In addition, the size distribution of all formulation nanogels did not significantly change after 30 days of storage at 4 °C (Table 1), suggesting that nanogels possess excellent physical stability.

### 2.5. Encapsulation Riboflavin in Modified Soy Protein/Dextran Nanogels

The FT-IR spectrum of the NGs, pure riboflavin, and the riboflavin-loaded NGs are shown in Figure 3. A series of bands were observed on the spectrogram of riboflavin, one for hydrogen bonded O–H stretch (3419 cm^−1^), one for carbonyl stretching vibration of the isoalloxazine ring (1728 cm^−1^), one for amide I C=O stretching (1628 cm^−1^), one for C=N stretching vibrations (1553 cm^−1^), and several others in the wave number range of 1300–1200 cm^−1^ indicating C–N stretching and N–H deformation [19]. All of these peaks existed in the spectrum of riboflavin-loaded nanogels with greatly reduced intensity, indicating some interaction between riboflavin and nanogels and substantial encapsulation of riboflavin in modified soy protein/dextran nanogels. Especially, all characteristic peaks of riboflavin were not detected in the spectra of riboflavin-loaded NG2 and NG3 (the broad band at ~3420 cm^−1^ and two medium intensity peaks at 1650 and 1542 cm^−1^ in the spectrum loaded NGs were derived from that of SPI instead of riboflavin). The disappearance of these characteristic peaks indicated successful encapsulation of riboflavin in the polymeric matrix of NG2 and NG3. On the other hand, the absorption bands of the -OH groups of riboflavin overlapped with the –NH stretching bands and also shifted to a lower frequency, which might be due to the molecularly dispersed riboflavin associating with NGs via hydrogen bonding. Moreover, there was a small decrease in the stretching vibration bands such as –C=O and –NH_2_. This information suggested that the carboxyl groups of riboflavin might have interacted with amino groups of soy protein through electrostatic interaction which also contribute to the driving force. 

The encapsulation efficiency (EE), loading capacity (LC) and size of riboflavin-loaded nanogels in function of the different final riboflavin concentration were shown in Table 2. The different modified method affected the loading content of riboflavin. With increasing riboflavin concentration, loading content of riboflavin in NG1 increased, but loading content of loaded NG2 and NG3 increased and then decreased. The maximum EE of riboflavin in NG2 and NG3 were 60.1% and 65.9%, respectively, which was obtained at 250 μg/mL of riboflavin concentration. In contrast, the drug loading capacity of all formulation nanogels showed a linear increase with the added riboflavin concentration. As shown in Table 1, the highest EE and LC of riboflavin was found in NG3, followed by NG2, and the least was NG1. These results further confirmed the aforementioned TEM images and ζ-potential analysis and indicated that modified soy protein/dextran nanogels had a compact core-shell-like structure, particularly for NG3. Meanwhile, the riboflavin encapsulation did not influence the particle size of the NGs. Besides, the particle size of riboflavin-NGs (NG2 and NG3) was slightly smaller than NGs, perhaps because the riboflavin increased the degree of nanogels compaction. The exact reason needs to be further investigated. Based on a balanced consideration of application essentials, e.g., small size, narrow size distribution, higher EE and LC, 250 μg/mL was believed to be the optimal concentration to prepare riboflavin-loaded nanogels.

### 2.6. Riboflavin Release from Modified Soy Protein/Dextran Nanogels

**Figure 10:**
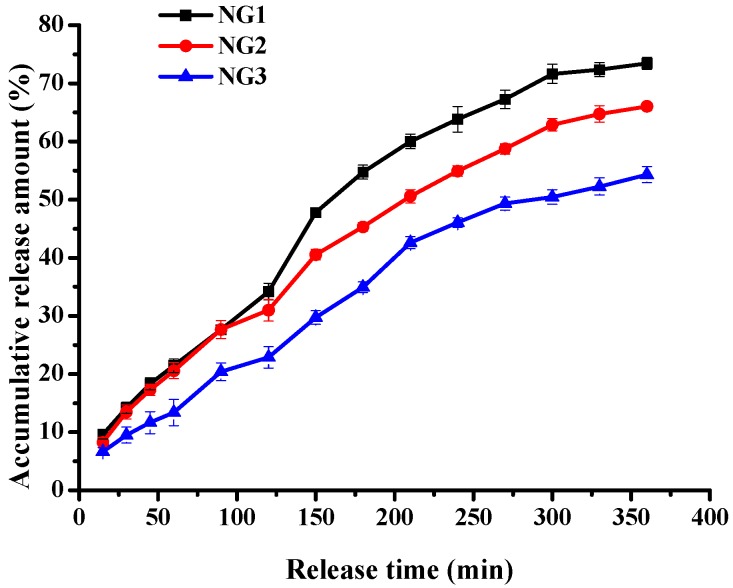
Release of riboflavin in simulated gastric and intestinal fluid from NG1, NG2 and NG3. The data with different lowercase letters in the same test are significantly (*p* < 0.05) different.

The average residence time in the gastrointestinal system is different after oral administration, which is approximately 2 h in the stomach, 4 h in the small intestine [20]. The *in vitro* release behavior of riboflavin from modified soy protein/dextran nanogels was evaluated under simulated gastrointestinal tract (GI) condition. Release profiles for all formulations are reported in Figure 10 and show the significant difference between all formulation nanogel samples incubated in simulated gastric fluid (SGF) and simulated intestinal fluid (SIF). The release of riboflavin from modified soy protein/dextran nanogels was higher at SIF than at SGF. That is, less than 34% of riboflavin was released from all formulation nanogels at SGF, whereas nearly 68% of the initial riboflavin content had been released 4 h after changing the medium to SIF. This was because hydrogen bonding and electrostatic interaction between the nanogels and riboflavin might be resistant to degradation in SGF. The initial burst release was not observed for all formulations under SGF and SIF, indicating that the release of riboflavin could be controlled and sustained in modified soy protein/dextran nanogel form. The NG3 showed the lowest release rates. Namely, the accumulated amount of riboflavin released from NG3 was <23% in the SGF, whereas it was 54.32% in the SIF. This could be due to the formation of a tighter core-shell structure. These results indicated that the modified soy protein/dextran nanogels were stable in simulated gastric fluid, and it reduced the undesirable side effects of riboflavin on the stomach. The *in vitro* evaluation suggested that the modified soy protein/dextran nanogels exhibited notably sustained release behaviour in the simulated medium of the gastrointestinal tract.

**Figure 11:**
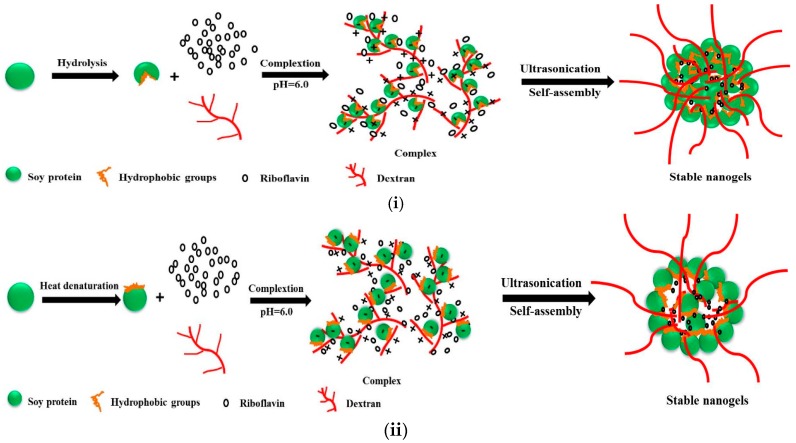
Diagrammatic depiction of modified soy protein/dextran nanogels formation. (**i**) illustration of the fabrication process of NG3; (**ii**) illustration of the fabrication process of NG3.

According to the above discussion, the proposed model of the modified soy protein/dextran nanogels formation pathway and *in vitro* delivery processing was illustrated in Figure 11. Heat denaturation and hydrolysis could result in the change of the original conformation of soy protein and the exposure of hydrophobic groups to the surface of the molecules [21,22]. Herein, the hydrophobic interactions among soy protein molecules might be enhanced during self-assembly between soy protein and dextran which changed from chains to near spherical aggregates gradually. We therefore speculated that hydrophobic and electrostatic interactions might be very important in the formation of nanogels and gelation of soy glycinin, which was almost the same as previous report of chitosan/ovalbumin and soy protein/soy polysaccharide nanogels [4,6]. As compared to hydrophobic and electrostatic interactions, hydrogen bonds seemed to play a relatively minor role in the stabilization of the nanogels structure. This kind of structure might be related to the pH stability of the nanogels. Furthermore, from the *in vitro* release behavior, we can make out that protonated riboflavin could be absorbed to dextran with longer chains by electrostatic interaction, and riboflavin could interact with modified soy protein by hydrogen bonding. Finally, the electrostatic and hydrogen bonding of modified soy protein, dextran and riboflavin can induce effective self-assembly. Riboflavin was loaded to modified soy protein/dextran nanogels either by adsorption to the surface of nanogels or by incorporation into the particle matrix. As a result, the riboflavin encapsulation efficiency was higher. The release rate of modified soy protein-dextran nanogels was relatively slow under simulated gastrointestinal tract (GI) conditions, which may be attributed to drug diffusion and the swelling/degradation of the biopolymer. Moreover, soy protein hydrolysates contains more hydrophobic protein region than heat denatured protein, which indicated that more hydrophobic protein on the dextran in the NG3 can take part in the gelation with the soy protein and form a closer structure.

## 3. Experimental Section

### 3.1. Materials

SPI was obtained from Wonderful Tech. Co. (Shandong, China), containing (on dry basis) 6.5% moisture, 1.0% ash, 0.2% lipid, and 90.2% protein (determined by Kjeldahl method, N × 6.25). Dextran (MW: 60,000–90,000) was purchased from Chanshou Biological Co., Ltd. (Jiangsu, China). Alcalase was purchased from Novo Nordisk’s Enzyme Business (Wuxi, China). Riboflavin (MM: 376.36; purity ≥ 98%) were purchased from Sinopharm Chemical Reagent Co., Ltd. (Shanghai, China). The other solvents/chemicals used were of analytical grade and obtained from Shanghai Chemical Reagent Co., Ltd. (Shanghai, China). All materials were used without further purification. Deionized water was used in all experiments.

### 3.2. Preparation of Complex Nanogels

Three sets of complex nanogels were prepared. In the first set, the soy protein dispersion was prepared by dispersing soy protein powder in deionized water (2%, *w*/*v*) and stirring at room temperature for 3 h. The soy protein dispersion was left for 12 h at 4 °C to allow complete hydration. Afterward, the dispersion was centrifuged at 8000× *g* for 15 min to remove insoluble matter. The dextran was dissolved in distilled water (2%, *w*/*v*) with slightly magnetic stirring for 3 h at room temperature. Then, the resultant soy protein solution was dropwise added into the dextran solution with vigorous magnetic stirring with weight ratios of 1:1. After the pH was adjusted, the obtained mixtures were ultrasonicated at 300 W for 70 min. The resultant nanogels (NG1) solution was either kept at 4 °C or freeze-dried.

In the second set, the soy protein dispersion (2%, *w*/*v*) was heated in a water bath while stirring at 60 °C for 30 min. After the heat treatment, samples were immediately cooled to room temperature and then mixed with dextran solution and ultrasonicated as in the first set. The resultant nanogel was referred to as NG2.

In the third set, soy protein hydrolysate was prepared with Alcalase using a pH-stat method. Both soy protein and Alcalase were dissolved in 0.2 M phosphate buffer solution (pH 9.5) to give a starting protein concentration of 7% (*w*/*v*) and the ratio of enzyme to substrate was 2%.The hydrolysis was conducted at pH 9.5 in a water bath shaker at 55 °C for 40 min, which was stopped by heating in boiling water for 15 min. After the hydrolysate was cooled to room temperature and centrifuged at 8000× *g* for 15 min, the supernatants were then collected. The resultant solution (NG3) was dropwise added into the dextran solution and ultrasonicated as in the first set.

The nanogels solutions containing designed pH values and NaCl concentrations were stored at 4 °C to investigate the stability. The resultant nanogel solution was adjusted to different pH values (2–10) with 0.5 mol/L NaOH or HCl solution, and then 3 mol/L NaCl solution was added to reach a 0.15 mol/L final NaCl concentration.

### 3.3. Riboflavin Encapsulation

Riboflavin was dissolved in water at the concentration of 1.0 mg/mL. Riboflavin solution was then added into the soy protein solutions, heat treated protein solution, soy protein hydrolysates, respectively. The obtained mixture was dropwise added into dextran solution to get a final riboflavin concentration of 50, 100, 150, 200, 250, 300, 350 g/mL, respectively. The following process was the same as the fabrication of NGs. The obtained riboflavin-loaded NGs were centrifuged at 4000× *g* for 30 min in a refrigerated centrifuge. Riboflavinin the percolated solutions was determined by a UV-vis spectrophotometer (Shanghai JINMI Science Instrument Co. Ltd., Shanghai, China) at 446 nm. The encapsulation efficiency (EE) and loading capacity (LC) were calculated as follows:

Encapsulation efficiency (EE) = (total amount of riboflavin − unloaded riboflavin)/total amount of riboflavin × 100%

Loading capacity (LC) = (total amount of riboflavin − unloaded riboflavin)/ total weight of nanogels × 100%

### 3.4. Characterization of Blank Nanogels and Riboflavin-Loaded Nanogels

Turbidity measurements were carried out at 600 nm by using a UV-vis spectrophotometer (Shanghai JINMI Science Instrument Co. Ltd.) according to the method of Chen *et al.* [23]. The average particle size of complex nanogels were determined by using a Zetasizer Nano-ZS instrument (Malvern Instruments, Worcestershire, UK).The morphological characteristics of blank NGs were analyzed by using a transmission electron microscope (JEM-2100F, JEOL, Tokyo, Japan) according to the method of Pujana *et al.* [24]. X-ray photoelectron spectroscopy (XPS) was performed on Krato Axis Ultra DLD spectrometer. The samples were prepared by centrifuging the nanogels solution at 4000× *g* for 30 min and then freeze-dried. The elements of soy protein, heat treated soy protein, soy protein hydrolysate and complex nanogels surface were identified according to the specific binding energy (eV). FTIR spectra were measured on a FT-IR spectrometer (NICOLET NEXUS470, DTGS, Thermo Scientific, Waltham, MS, USA). Diffusive reflectance of the IR was measured using lyophilized samples diluted with potassium bromide (KBr) powder of spectroscopic grade by one-tenth and recorded with an average of 32 scans at a resolution of 4 cm^−1^. Background noise was corrected with pure KBr data. Nanogel morphologies were analyzed by SEM, by placing a drop of nanogel sample on carbon stickers on aluminum stubs, drying and coated with gold, prior to visualization.

### 3.5. In vitro Release Studies

Briefly, an aliquot of riboflavin loaded nanogels was taken into a dialysis bag (3500 MWCO) and then suspended in100 mL simulated gastric fluid (SGF, pH 1.2, HCl added pepsin)for 2 h at 37 °C. And then, this dialysis bag with nanogels was placed in 100 mL simulated intestinal fluid (SIF, pH 7.4, PBS added trypsinase) and further incubated in a water bash shaker at 150 rpm for 4 h at 37 °C. At certain time intervals, 2 mL of medium was collected and the same amount of fresh release fluid was supplemented every time. The release amount of riboflavin was determined by UV-spectrophotometry at 446 nm. UV absorption spectra of the FA dissolved at simulated gastrointestinal fluid directly and the released FA from complex nanogels in simulated gastrointestinal fluid were recorded on a spectrophotometer (Shanghai JINMI Science Instrument Co. Ltd.) to calculate the riboflavin concentration in the ultrafiltrate after deduction of the background. 

### 3.6. Statistical Analysis

All measurements were performed in triplicate. The results obtained are presented as mean ± standard error of the mean (SEM). Data were processed by analysis of variance using the Origin 7.5 software (Origin Lab Corp., Northampton, MA, USA), and *p* < 0.05 was considered as statistical significant.

## 4. Conclusions

A simple self-assembly method induced by ultrasonication was used to fabricate modified soy protein/dextran nanogels. NG formation was confirmed by FTIR spectroscopy and XPS analysis. Compared with soy protein-dextran nanogels (NG1) with a hydrodynamic diameter of about 138 nm, the modified soy protein/dextran nanogels were near spherical in shape with core-shell structures, as evidenced by XPS and TEM studies and ζ-potential analysis, and the hydrodynamic sizes of nanogels (NG2 and NG3) were about 57nm and 66nm, respectively. Moreover, these modified soy protein/dextran nanogels were very stable against long-term storage at 4 °C, pH (2–10) and low ionic strength (<0.15 mM) changes, whereas NG1 exhibited susceptible to acidity. Riboflavin was successfully loaded into stable nanogels via electrostatic interactions and hydrogen bonding between modified soy protein/dextran and riboflavin. Notably, modified soy protein/dextran nanogels prepared using riboflavin concentration of 250 μg/mL achieved high loading efficiency around 10.4% and 12% and small size 53.6 and 64.9 nm, respectively, but NG1 obtained a loading efficiency about 7% and size of 143.3 nm. Besides, the *in vitro* release study of riboflavin in SIF and SGF showed that all nanogels obviously have a sustained release effect which indicated that the structural relaxation was comparable to diffusion, and the release in SGF was slower compared with in SIF, which could protect the riboflavin in the stomach and ensure it released in the intestines. Furthermore, among the three tested complex formulas, NG3 resulted in a least amount of released riboflavin, followed by NG2, and more released in NG1. Therefore, this feature makes modified soy protein/dextran nanogels a promising carrier for controlled-release bioactive substance delivery systems.

## Figures and Tables

**Table 1:** Effect of storage time (30 days) on the particles sizes of NG1, NG2 and NG3; the results are displayed as the mean ± standard deviation (*n* = 3).

Storage Time (Days)	Particle Size (nm)		
	NG1	NG2	NG3
0	138.3 ± 3.9	57.2 ± 0.7	66.8 ± 0.6
30	141.3 ± 5.2	60.7 ± 1.8	69.4 ± 1.2

**Table 2:** Drug encapsulations, loaded efficiencies and particle sizes of the NGs in different riboflavin concentrations; the results are displayed as the mean ± standard deviation (*n* = 3).

Riboflavin Concentration (μg/mL)	NG1			NG2			NG3		
	LC (%)	EE (%)	Particle Size (nm)	LC (%)	EE (%)	Particle Size (nm)	LC (%)	EE (%)	Particle Size (nm)
50	1.9 ± 0.1	29.8 ± 0.6	152.7 ± 4.2	3.9 ± 0.2	38.9 ± 1.6	42.8 ± 2.6	4.2 ± 0.4	43 ± 1.8	49.1 ± 3.4
100	2.6 ± 0.2	35.1 ± 0.5	147.3 ± 5.1	4.7 ± 0.2	45.5 ± 2.2	47 ± 3.7	5.9 ± 0.2	47.7 ± 2.7	52.2 ± 2.7
150	3.8 ± 0.4	41.7 ± 1.6	141 ± 4.5	6.2 ± 0.8	49.3 ± 3.2	45.9 ± 3.5	7.5 ± 0.7	54.9 ± 3.4	57.1 ± 2.3
200	4.7 ± 0.3	46.6 ± 2.3	138.6 ± 5.6	8.8 ± 0.9	55.4 ± 2.6	50.8 ± 2.2	9.9 ± 0.5	58.7 ± 3.8	61.8 ± 4.1
250	7 ± 0.4	55.6 ± 0.9	143.4 ± 3.3	10.4 ± 0.5	60.1 ± 2.1	53.6 ± 1.8	12 ± 0.9	65.9 ± 1.2	64.9 ± 2.5
300	9.6 ± 0.8	56.9 ± 1.0	144.7 ± 3.6	12.3 ± 0.6	57.4 ± 1.4	56.6 ± 2.4	13.8 ± 1.1	60.1 ± 0.9	65.2 ± 1.9
350	11 ± 0.7	58 ± 0.8	148.2 ± 2.5	14.6 ± 0.8	54.6 ± 1.8	55.2 ± 2.8	15.9 ± 0.4	58.8 ± 1.1	65.8 ± 2.6
